# Host-Microbiome Interactions Mediated by Phenolic Metabolites in Chronically Critically Ill Patients

**DOI:** 10.3390/metabo11020122

**Published:** 2021-02-20

**Authors:** Ekaterina Chernevskaya, Natalia Klimenko, Alisa Pautova, Irina Buyakova, Alexander Tyakht, Natalia Beloborodova

**Affiliations:** 1 Federal Research and Clinical Center of Intensive Care Medicine and Rehabilitology, 25-2 Petrovka Str., 107031 Moscow, Russia; alicepau@mail.ru (A.P.); bivbiv72@yandex.ru (I.B.); nvbeloborodova@yandex.ru (N.B.); 2 Atlas Biomed Group—Knomics LLC, 31 Malaya Nikitskaya Str., 121069 Moscow, Russia; natasha.klmnk@gmail.com (N.K.); a.tyakht@gmail.com (A.T.); 3 Center for Precision Genome Editing and Genetic Technologies for Biomedicine, Institute of Gene Biology Russian Academy of Sciences, 34/5 Vavilova Str., 119334 Moscow, Russia

**Keywords:** host-microbe interactions, microbiome, metabolomics, gut bacteria, 16S rRNA, GC-MS, microbial metabolites, neurological injury, gut-brain axis

## Abstract

The community structure and metabolic potential of gut microbiome is not well investigated, especially in chronically critically ill patients with prolonged dependence on support systems after severe brain disorders. Microbial phenolic metabolites can target the brain function by the direct and indirect modulation of inflammation. The aim of this study was to investigate the features of the gut microbiota and profile of certain metabolites in the progression and reversibility of neurological disorders in chronically critically ill patients. Fecal samples were collected in dynamics from such patients (*n* = 44) and analyzed using 16S rRNA sequencing. Serum microbial and mitochondrial metabolites were measured by GC-MS and compared with the biomarkers and clinical neurological scores. The identified associations between specific bacterial taxa in fecal samples, neurological status and serum levels of metabolites suggest that impacts on specific members of the gut microbiota and their metabolism might be a promising tool for regulating brain function in future.

## 1. Introduction

Over the past decades, advances in therapeutic techniques have substantially improved the survival of patients after acute events, and simultaneously expanded the population of patients with prolonged dependence on life support systems. This gave rise to the concept of chronic critical illness (CCI) [1]. CCI criteria include at least 2 weeks in an intensive care unit (ICU), prolongated (14–28 days) mechanical ventilation, persistent inflammation, immunosuppression, hypermetabolism and hypercatabolism and increased susceptibility to infection [2].

Most surviving patients after severe brain injury meet CCI criteria. They are compromised by the risk of the CCI-attributed neurological dysfunction aggravated by their condition. It may be caused by the functional or metabolic impairment mediated by pro- or noninflammatory factors, including microglia hyperactivation, blood brain barrier disruption and altered neurotransmission [3].

Taxonomic changes and dysfunction of the gut microbiota may be important factors in CCI. The gut microbiota has the ability to affect the central nervous system (CNS) function [4,5], making the gut microbiota a promising target for therapeutic approaches. A number of studies of the microbiota of critically ill patients is growing [6,7]. The gastrointestinal tract is known to be involved in the development and progression of organ failure in critically ill patients [8,9,10]. CCI patients have severe metabolic disorders leading to the protein-energy malnutrition and gastrointestinal tract comorbidities caused, among other things, by the nasogastric nutrition [11], which may be the main causes of the dysbiosis in such patients [12]. The composition of the gut microbiota in patients is quite variable and undergoes dynamic changes, and not only taxonomic but also metabolic microbial profile should be considered. The gut microbiota produces a plethora of metabolites including, particularly, the short-chain fatty acids, secondary bile acids, neurotransmitters, phenylcarboxylic and hydroxy acids [13,14].

The significant role of such microbial metabolites as phenylcarboxylic acids (PhCAs) among many groups of studied low-molecular weight compounds in blood of septic patients have been demonstrated [13]. PhCAs are the products of the degradation of phenylalanine, tyrosine and polyphenols produced by the diverse bacterial taxa including, for example, *Staphylococcus aureus, Klebsiella pneumonia, Acinetobacter baumanii, Escherichia coli* and *Pseudomonas aeruginosa*. [15,16]. The following PhCAs are potentially involved in the pathological processes: phenyllactic acid (PhLA), phenylpropionic acid (PhPA), phenylacetic acid (PhAA), p-hydroxyphenylacetic acid (p-HPhAA) and p-hydroxyphenyllactic (p-HPhLA) acid. These compounds affect the mitochondrial functions and the phagocyte activity of neutrophils [17,18] and reflect the severity of the bacterial inflammatory process [19]. Additionally, their levels are correlated with Acute Physiology and Chronic Health Evaluation II (APACHE II) and Sequential (Sepsis-related) Organ Failure Assessment (SOFA) scales [20]. The pilot study on patients with CCI showed a qualitative and quantitative imbalance of microbial metabolites associated with changes in the microbiome [21]—serving a basis for the presented study.

Since microbial metabolites are linked to the mitochondrial dysfunction [13], which in turn leads to the neurodegenerative processes [22], in the present study we evaluated serum concentrations of microbial metabolites along with the mitochondrial metabolites.

The aim of this study was to investigate the features of the gut microbiome and certain microbial and mitochondrial metabolites in the progression and reversibility of neurological disorders in chronically critically ill patients.

## 2. Results

### 2.1. Metabolic and Microbiome Differences between Healthy Subjects and CCI Patients

**Figure 1:**
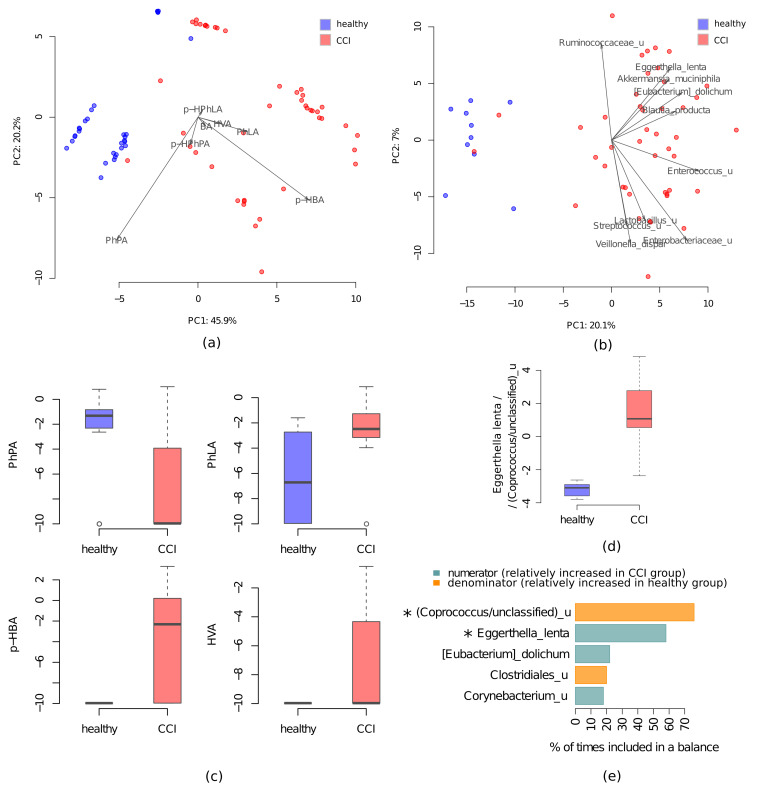
Comparison of the gut microbiome and serum metabolites between the healthy subjects and CCI patients. (**a**) PCA of metabolic profiles from the CCI patients (red dots) and healthy subjects (blue dots). (**b**) PCA of the CCI patients (red dots) and healthy subjects (blue dost) microbiome samples after clr transformation (Aitchison distance). Arrows show the top 10 taxa in terms of the variance explained by the axes (the arrow length is proportional to the explained variance, and the arrow angle reflects the distribution of this variance between the axes). (**c**) The levels of metabolites differentially abundant in the healthy subjects and CCI patients, the circles denote boxplot outliers (**d**) Values of microbiome balance at the level of species. (**e**) Reproducibility barplot for the 3 most frequent balance members during the cross-validation procedure: the blue bars correspond to the numerator members (part of the balance higher in patients) and orange—to the denominator members (part of balance higher in healthy subjects). Asterisks (*) denote taxa which are considered reliably associated with the group according to the balances-based approach (reproducibility of bacterial taxa > 50% and R^2^ > 0.2).

Serum and fecal samples were collected from each CCI patient and healthy subject. Overall, we identified 75 bacterial taxa and 4 PhCAs that were differentially abundant between the CCI patients and healthy subjects. The latter groups formed two distinct clusters as a results of each of the metabolic and taxonomic principal component analyses (PCA) (Figure 1a,b). 

Among the phenolic metabolites, serum concentrations of PhLA, p-HBA and HVA were higher in the CCI patients, while PhPA concentration was higher in healthy controls (FDR < 0.05, Table 1, Figure 1c). The serum levels of fumaric acid (FA) in the CCI patients <1 µM in most cases (in 57 out of 77 samples). The levels of mitochondrial FA and succinic acid (SA) were lower than those in the control group; the SA levels were > 30 µM only in 4 cases (5%). The highest and the lowest values are not shown in Table 1 as they are outside of the IQR (25–75%). Additionally, for the CCI patients, proinflammatory interleukin-6 (IL-6) along with the bacterial infection (procalcitonin, PCT) and neurological (S100 protein) biomarkers are presented in Table 1. IL-6 levels were higher in all CCI patients, while the other biomarkers did not deviate from their reference values. We observed moderate Spearman’s correlations between the levels of PCT and SA (r = 0.38) and FA (r = 0.36).

Almost all (73 of 75 positive associations; 97%) differentially abundant taxa had higher levels in the healthy subjects, and their list included commensals like unclassified species from *Coprococcus/unclassified* genus, *Roseburia/Blautia* genera and *Faecalibacterium prausnitzii* (Supplementary Table S1). Positive associations with the CCI patients were observed only at relatively large taxonomic ranks for *Bacilli* and *Lactobacillales*. 

The results of the balances-based approach application [24] showed the existence of a bacterial balance that can predict the patient group (CCI versus subjects) by microbiome composition (AUC = 0.95). The two-components balance was selected as the optimal by the algorithm. The main predictors of the healthy group were unclassified species from *Coprococcus/unclassified* genus (the reproducibility of the taxon was 76%) (Figure 1d,e). The level of *Eggerthella lenta* (the reproducibility: 58%) was the main predictor of the CCI group. Other candidate taxa showed low reproducibility (< 50%) (Figure 1e).

Thus, the CCI patients demonstrated significant disruption in both microbiome composition and serum levels of the microbial and mitochondrial metabolites compared to the healthy subjects.

### 2.2. Metabolic and Microbiome Differences between the CCI Patients with Positive and Negative Clinical and Neurological Dynamics

**Figure 2:**
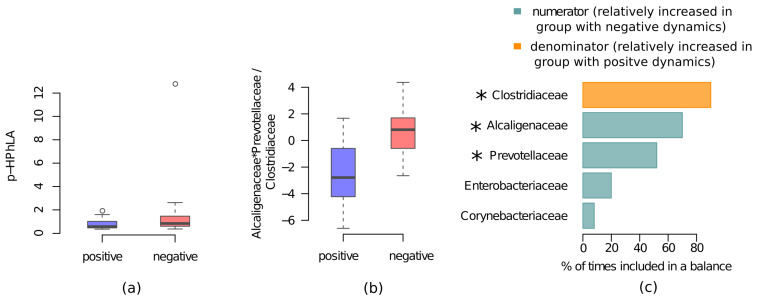
Associations between metabolic and microbiome profiles and positive and negative dynamics in the CCI patients. (**a**) The p-HPhLA levels in patients with positive and negative dynamics, the circle denotes boxplot outliers. (**b**) Values of microbiome balance at the level of families which was selected as the optimal one to distinguish the patients with positive and negative dynamics. (**c**) Reproducibility barplot for the 3 most frequent balance members during the cross-validation procedure (blue bars correspond to numerator members (part of balance more abundant in patients with negative dynamics) and orange-to the denominator members (part of balance more abundant in patients with positive dynamics)). Asterisks (*) denote taxa which are considered to be reliably associated with the group according to the balances-based approach (reproducibility of bacterial taxa > 50% and R^2^ > 0.2).

As the comparison of healthy subjects’ and CCI patients’ microbiome, we used two approaches to investigate associations between the patients’ microbiome and clinical dynamics: the approach focused on individual taxa abundances and the balances-based approach. Individual taxa analysis considered all time points and revealed 1 significant association (FDR < 0.05): the *Clostridiaceae* family was enriched in patients with positive dynamics. The balances-based approach considered the last time point per patient; it was performed at the taxonomic level of the family and demonstrated AUC = 0.76. The best balanced included three taxa: *Alcaligenaceae* and *Prevotellaceae* as numerator (associated with negative dynamics) and *Clostridiaceae*—as denominator (associated with positive dynamics). All three taxa showed relatively high reproducibility, achieving the highest value for the *Clostridiaceae* (90%) (Figure 2). 

We checked if the dynamics could be predicted by the alpha diversity of the community (Chao1 index) across all measured time points with the adjustment for the subject ID: no significance was found (*p* = 0.82). We also analyzed how metabolite levels at all time points were associated with the positive and negative dynamics, but no significant results were observed neither. 

As for the changes between the first and last time points, neither the metabolite level changes nor microbial abundance changes were significantly associated with positive/negative dynamics (for patients with at least 2 time points, *n* = 26, ANCOVA analysis).

We analyzed microbial metabolites and microbiome parameters associated with neurological scales (across all time points, adjusted for the patients ID, Table 2). Negative associations of PhPA with the Rankin scale and positive—with the Glasgow Coma Scale (GCS) were observed (FDR = 0.0336 for both, linear model coefficient −1.4 for the Rankin and 0.65 for GCS). The taxa related to *unclassified/Clostridiaceae/Lachnospiraceae* and *Ruminococcus bromii*/*unclassified* were associated negatively with Rankin scale and positively—with the Rivermead Mobility Index (FDR < 0.05). Negative association was also observed between species from *Coprococcus*/*unclassified* genus and *Roseburia* genus with the Rankin scale (FDR < 0.05). The alpha diversity was not significantly associated with the neurological state.

**Figure 3:**
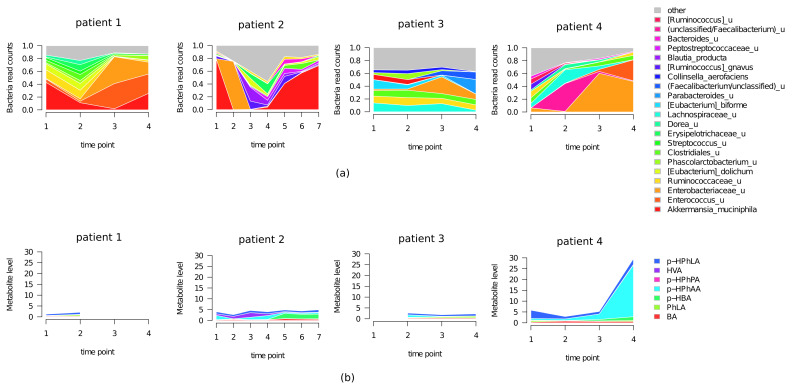
Temporal dynamics (time points) of microbiome composition (**a**) and metabolic (**b**) profile for patients 1, 2 with positive and patients 3,4 with negative dynamics.

The examples of temporal dynamics for two patients with positive dynamics (patients 1 and 2) and two patients with negative dynamics (patients 3 and 4) are shown (Figure 3). The figure clearly illustrates that the patterns of microbiome changes are patient-specific ones.

The gut microbiome profile of all CCI patients (patients 1–4) was characterized by rapid individual changes. Fecal samples of patients 1 and 2 with positive dynamics differed from ones of the patients 3 and 4 with negative dynamics by the prevalence of anti-inflammatory taxa such as *Akkermansia_muciniphila*, *Ruminococcaceae_u* and *Clostridiales_u*. Fecal samples of patients 3 and 4 were characterized by the prevalence of proinflammatory *Enterobacteriaceae_u* and *Enterococcus_u*.

The total level of the PhCAs in serum samples of patients 1–3 was within the normal level (<5 µM) previously reported for healthy subjects [23]. Healthy subjects were characterized by the prevalence of BA, PhPA and smaller values of sepsis-associated hydroxylated metabolites PhLA and p-HPhLA. All CCI patients (pts 1–4) were characterized by the absence of PhPA and small levels of BA contrasting to the prevalence of sepsis-associated PhLA, p-HPhAA and p-HPhLA. However, low level of different sepsis-associated metabolites in patients 2 indicated the presence of moderate inflammation which was confirmed by the level of IL-6 < 25 pg/mL. The patients 4 was also characterized by moderate inflammation and low level of sepsis-associated metabolites and IL-6 in points 1–2, but with subsequent onset of the multiple organ failure, accompanied by rapid increase of PhCAs (up to 30 µM) and IL-6 (up to 200 pg/mL) in the points 3 and 4, eventually leading to lethal outcome.

### 2.3. Association between Metabolites, Biomarkers and Microbiome

We calculated cross-correlation between metabolites levels and microbial taxa abundance for the CCI patients. Analysis of individual taxa did not reveal significant associations (performed across all time points with adjustment for the subject ID). The balances-based approach at the level of species showed several reliable associations for the last time point (Table 3). Moreover, there was a positive link between the alpha diversity and PhPA level (FDR = 0.0398). 

### 2.4. Associations of Taxa and Metabolites with the Therapy

We evaluated the effect of antibiotics use, nutrition type, diagnosis and infectious complications (Table 4) on the gut microbiota and metabolites. A low sample size hampered the inclusion of the effect of these variables altogether in the linear models. Thus, we investigated how microbiome and metabolites were affected by these parameters separately. In this analysis, we utilized all measured time points per patients with the adjustment for the patient ID. None of the metabolites were associated with the clinical data or diagnosis. The taxa related to *unclassified/Clostridium* were negatively associated with the antibiotic intake (FDR < 0.05). The antibiotic intake also was negatively associated with the alpha diversity on the edge of significance (FDR = 0.0529). Diagnosis and infectious complications of the CCI patients did not affect the microbiota composition. The *[Eubacterium] dolcium* and *Ruminococcus bromii/unclassified* were significantly enriched in the microbiome of the patients with the nasogastric tube or gastrostomy compared to those with oral enteral nutrition (FDR < 0.05), but this finding requires further confirmation in clinical studies with larger group sizes. 

## 3. Discussion

In this study, we found that the abundance of the specific gut microbial taxa along with the serum levels of certain microbial and mitochondrial metabolites in the CCI patients were significantly different from the ones of the healthy subjects, independent oftheir neurological dynamics. The features of the clinical course allowed us to include heterogeneous patients in the studied groups, regardless of their primary nosological form of severe brain damage.

Gut microbiota dysbiosis in patients with CCI is characterized by prevalence of *Proteobacteria* and Gram-positive opportunistic bacteria, such as *Enteroccocus* and *Streptococcus* coupled with the reduced abundance of commensal microorganisms. The balance-based approach showed that the main predictors for the CCI group were *Eggerthella lenta* (58% reproducibility), while for the healthy group there were unclassified species from *Coprococcus/unclassified* genus, similarly to the analysis of individual taxa. These facts are consistent with the findings of the previous studies of the gut microbiota in critically ill patients with brain injury [25,26]. The significant dysbiosis in stroke survivors manifested as the dominance of the short-chain fatty acid producers, including *Akkermansia*, was correlated with their clinical outcomes [27]. However, the authors of the cited study evaluated the composition of the gut microbiota within the first 48 h after stroke, and we have not found any publications describing microbiota composition in chronically ill neurological patients; these facts suggest the novelty of our findings. Previously, the significant differences for four genera (*Prevotella*, *Klebsiella*, *Streptococcus* and *Clostridium XI*) were found to be associated with some neuropsychiatric disorders [28,29]. The *Eggerthella lenta* is an emerging and uncommon human anaerobic opportunistic pathogen under-recognized due to the limitations of phenotypic identification that can lead to the severe bloodstream infection [30]. Noteworthy, the conversion of dopamine to m-tyramine occurs under the action of molybdenum-dependent dehydroxylase from *Eggerthella lenta* in the gut [31], which might play a role in the neurological dysfunction.

We identified the associations between several gut microbial taxa and neurological scales (Table 2). The *Ruminococcus, Roseburia* and *Clostridiaceae* were positively correlated with the improvement of the neurological status-specifically, by manifesting positive association with the Rivermead Mobility Index and negative association-with the Rankin scale. Among them, *Roseburia* known for its metabolic capacity of producing short-chain fatty acids has been reported to be depleted in fecal samples of sarcopenic subjects [32]. In addition, we found that the increased abundance of *Clostridiaceae* in the gut was associated with the positive neurological dynamics in the CCI patients. Previously, it has been shown that the arthritis phenotype is also characterized by an increase in the number of *Clostridiaceae* [33]. The *Clostridium* bacteria are among the phylotypes able to metabolize aromatic amino acids, such as tyrosine [34]. The relative contribution of the increased activity of tyrosine degradation possibly connected with the increase of *Clostridium* among the patients should be further investigated on larger cohorts. Another interesting finding, *A. muciniphila* is best known for its potential anti-inflammatory properties [35]; nevertheless, this microorganism is also associated with the pro-inflammatory pathways and play an important role in the neurodegenerative diseases [36]. As shown earlier, the increased levels of this taxon can be observed in CCI patients [21]. However, we did not observe significant differences in patients with the positive and negative neurological dynamics, but *A. muciniphila* was abundant in a number of patients with positive dynamics, presumably indicating its important role in the neurorehabilitation. Overall, the described results provide additional support for the “gut-brain axis” concept. 

Three phenolic metabolites-PhLA, p-HBA and HVA-had higher levels in CCI patients, while only one (PhPA) had higher values in healthy controls, which resonates with the results of our pilot study [21]. The levels of two mitochondrial metabolites, SA and FA, were lower compared to the healthy subjects. Interestingly, the results in the CCI group are comparable to the previously obtained levels of these metabolites in patients with the early stage of sepsis [13]. The succinic acid (SA), a microbial intermediate in the citric acid cycle, does not accumulate in serum at any substantial levels under normal conditions. Elevated concentrations of SA concentrations in serum have been commonly reported in hypoxia, metabolic and inflammatory diseases [37]. At the same time, reduced levels of SA compared to reference values are not considered as clinically significant marker. However, two valeric acid esters in rats fed with high-fat diet have been shown to affect cecal microbiota composition and decrease the liver SA [38]. We have also found a positive association of low level of SA with taxa related to *Bifidobacterium*, which is relatively less abundant in the CCI patients, in turn, suggesting the importance of this finding as a reflection of the gut microbiota dysfunction. 

PhPA is the metabolite of the tyrosine and phenylalanine bacterial biotransformation [16]. The association between PhPA and *Coprococcus_u* (Table 3) is in line with the results of the study where this taxon was depleted in patients with depression and had the potential to the synthesis of important tyrosine metabolite (DOPAC, 3,4-dihydroxyphenylacetic acid) which is positively correlated with mental health [39]. PhPA association with commensal taxa abundance has been also observed in our previous study [21]. Moreover, PhPA was positively associated with GCS and negatively—with the Rankin scale, indicating the potential significance of the *Coprococcus* for the restoration of the level of consciousness.

The PCT levels were within the reference range in the CCI patients. At the same time, high levels of IL-6 were observed in all patients. Such levels of biomarkers indicate aseptic inflammation not associated with systemic bacterial infection. It has been shown that IL-6 levels reflect the degree of impairment of consciousness according to the GCS in the CCI patients with gut dysbiosis [40]. Here we did not find significant correlations between CCI patients with the positive and negative dynamics possibly due to the small number of patients. However, the connection between inflammation and gut dysbiosis is being actively discussed in literature, so IL-6 might be an effective marker of the gut microbiota dysfunction. This assumption can be illustrated by the results of animal study: the high-fiber diet has significantly reduced IL-6 in feces and serum, as well as affected gut microbiota by increasing the relative abundance of the anti-inflammatory bacteria, while decreasing the proinflammatory bacteria [41].

The enrichment of the commensal bacteria in CCI patients was associated with positive neurological dynamics. This is in agreement with the larger abundance of commensal bacteria observed in the healthy subjects compared to CCI patients described above. A recent report described the microbiome of patients with inflammatory bowel disease (IBD) and the disease-associated dysbiosis was characterized by depletion of commensal microbes, contrarily to the very few taxa significantly enriched in the patients [42]. We observed that the individual-specific profile of gut dysbiosis in the CCI patients (Figure 3) and microbiome/metabolome changes reflected the neurological dynamics.

**Figure 4:**
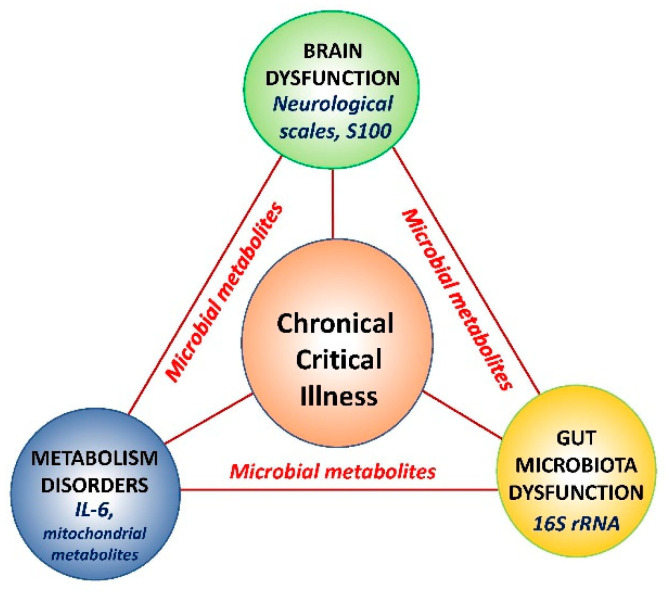
The schematic representation of the factors affecting the development of a chronic critical illness with the involvement of dysfunction of key organs—the brain and the gut (via gut microbiota), and the metabolic disorders, mediated by microbial metabolites.

To summarize, the gut microbiota of the CCI patients with brain dysfunction undergoes certain transformations and can be considered as a “damaged organ”, which is schematically shown in Figure 4. The study was limited by the small number of patients and biological samples and the influence of the factors affecting gut microbiota composition, such as various antibiotic and nutrition therapy was not fully clarified.

## 4. Materials and Methods

### 4.1. Study Design 

This prospective observational study was performed in the Department of Intensive Care at Federal Research and Clinical Center of Intensive Care Medicine and Rehabilitology, Moscow, Russian Federation. The local Ethics Committee approved the study (#2/19/2, 10/06/19) which was conducted in accordance with the ethical standards of the Declaration of Helsinki and formal consent for participation in this study was obtained from each patient or his/her legal representative.

### 4.2. Patients and Samples

Study Participants. We included adult (>18 years) patients admitted to the Department between June 2019 and December 2019. The following inclusion criteria were applied: patients with brain injury of various etiology due to the CCI criteria (at least 2 weeks in ICU and/or 14–28 days of mechanical ventilation, aged 18–75 years) and admitted to ICU for the intensive neurological care. Exclusion criteria: chemotherapy/hormonal therapy with steroids; suspected neurological infection, acute neurological disorders. 

The age range of the CCI patients (*n* = 44) was from 23 to 75 years (median: 50 (IQR: 31–64) years). The study group was balanced by gender: 23 (52%) females and 21 (48%) males. The pathologies recorded during the study included an acute cerebrovascular event (*n* = 16), severe traumatic brain injury (TBI) (*n* = 13), neurosurgery complications in the postoperative period (*n* = 12), anoxic brain injury after a successful cardiopulmonary resuscitation effort to reverse clinical death (*n* = 3). Some patients were on mechanical ventilation (*n* = 14) and some had a tracheostomy tube (*n* = 9). All patients were on enteral feeding; among them, over a half (*n* = 23) were fed through a nasogastric or gastrostomy tube. Antibacterial therapy was administered to patients (*n* = 13) who had urinary and/or lower respiratory tract infection. 

The neurological status of patients was assessed using the widely known scales: Glasgow coma scale (GCS), the National Institutes of Health Stroke Scale (NIHSS), the Rivermead Mobility Index Scale and the Rankin Scale. 

The Rankin Scale is used to measure the degree of neurological disability. It is widely used for the clinical outcome measure in the stroke clinical trials. The scale ranges from 1 to 5 where score 1 shows no significant disability and score 5 indicates severe disability, which requires constant nursing care and attention [43]

The Rivermead Mobility Index (RMI) is a hierarchical mobility scale used in neurological rehabilitation. It reflects to different aspects of mobility, with the sum scores ranging from 0 to 15, with higher scores indicating better mobility.

The Glasgow Coma Scale (GCS) is a clinical scale used to reliably measure a person’s level of consciousness after a brain injury. A person’s GCS score can range from 3 (completely unresponsive) to 15 (responsive).

The National Institutes of Health Stroke Scale, or NIH Stroke Scale (NIHSS) is a tool which is used to objectively quantify the impairment caused by a stroke. The maximum possible score is 42, which reflects no stroke symptoms; the minimum score is 0 and reflects a severe stroke.

A positive dynamic was defined as a decrease in the number of points by 1 or more on the NIHSS scale; a negative dynamic was defined as an increase in the number of points by one or more on the NIHSS scale or a lethal outcome. Characteristics of the two groups of CCI patients is demonstrated in Table 5. 

Blood (from a central venous catheter) and gut microbiome samples (feces) were collected simultaneously in the morning at regular intervals (once a week). Fecal samples were obtained by collecting a small amount of feces as a rectal swab and dissolved in 1 mL of sterile saline solution; after thorough mixing, it was divided into two Eppendorf tubes. All samples (serum and feces) were frozen and stored at −30 °C prior to analysis that included GC-MS, biomarkers and general clinical analyses, laboratory tests and sequencing. 

The total number of simultaneously taken fecal and blood samples was 83. In 18 patients, 1 sample was taken; in 16 patients—2 samples; in 10 patients—3 samples or more.

As a control group, we used previously obtained data on serum metabolites of 20 healthy volunteers without any signs of neurological or somatic pathology [23]. The age range of healthy subjects was from 25 to 64 years and the group was balanced by gender. Healthy controls did not have chronic liver and kidney diseases or clinical signs of acute inflammation; they all had a normal body temperature and normal levels of leukocyte, platelet count, hemoglobin, bilirubin, urea and creatinine, they did not receive any antimicrobial drugs for three months prior to sample collection.

### 4.3. Reagents and Chemicals

The 2,3,4,5,6-D5-benzoic acid (internal standard, ≥99 atom % D, ≥99%), benzoic acid (BA, ≥99.5%), phenylpropionic acid (PhPA, ≥99%), phenyllactic acid (PhLA, ≥98%), 4-hydroxyphenylacetic acid (p-HPhAA, ≥98%), 4-hydroxyphenylpropionic acid (p-HPhPA, ≥98%), homovanillic acid (HVA, ≥97%), 4-hydroxyphenyllactic acid (p-HPhLA, ≥97%), 3,4-dihydroxybenzoic acid (internal standard, ≥98%), succinic acid (≥99%), fumaric acid (≥99%), N,O-bis(trimethylsilyl)trifluoroacetamide (contains 1% trimethylchlorosilane, 99% N,O-bis(trimethylsilyl)trifluoroacetamide), hexane (≥97.0%) were obtained from Merck (Darmstadt, Germany); sulfuric acid, acetone, diethyl ether, sodium chloride were laboratory reagent-grade and obtained from Khimreactiv (Staryy Oskol, Russia).

### 4.4. Serum Sample Preparation and GC-MS Analysis

The blood serum samples were thawed at a room temperature prior to analysis. The GC-MS analyses were performed on a Trace GC 1310 gas chromatograph equipped with an ISQ LT mass spectrometer and AI 1310 autosampler using the capillary column TR-5ms (95% poly(dimethylsiloxane) + 5% phenyl polysilphenylene-siloxane phase, 30 m × 0.25 mm, df = 0.25 µm) obtained from Thermo Scientific (Thermo Electron Corporation, Waltham, MA, USA). The column flow was constant at 1.5 mL/min with helium as the carrier gas, split 1:10. The GC analysis was performed in 25 min with a starting oven temperature of 80 °C (hold time 4 min) and a single ramp of 10 °C/min to 250 °C (hold time 4 min). The injector temperature was 200 °C. Full-scan mass spectra were recorded with an *m*/*z* range of 50–480 in the electron-impact mode at 70 eV, using Xcalibur 2.2 software. The MS source was 200 °C and the GC-MS interface was kept at 250 °C. Scan rate was 3 scans/s; cathode delay time 4 min.

The conditions of liquid–liquid extraction of the microbial and mitochondrial metabolites extraction were previously described [23]. Briefly, an aliquot (200 µL) of serum and aliquots (100 µL) of aqueous solution of internal standards (2,3,4,5,6-D5-benzoic and 3,4-dihydroxybenzoic acids with a concentration of 4 ng/µL) were diluted with 600 µL of distilled water. Solid sodium chloride (0.3–0.5 g) and concentrated sulfuric acid (15 µL) were added for the protein precipitation. An extraction with diethyl ether was carried out (2 × 1 mL). The ether extract was evaporated at 40 °C and derivatized with N,O-bis(trimethylsilyl)trifluoroacetamide (20 µL, 80 °C, 15 min) to obtain volatile trimethylsilyl derivatives. The solution with trimethylsilyl derivatives was cooled at 5 °C for 30 min, diluted with 400 µL of n-hexane, and 2 µL of the final solution was injected into the GC-MS system.

Trimethylsilyl derivatives of the microbial and mitochondrial metabolites were identified using retention times and characteristic *m*/*z* values and their concentrations were calculated using the equations of linear functions which were previously described [13,23].

### 4.5. Microbiome Sample Preparation

Defrozen fecal solution (200 mL) was placed in the 2.0 mL tube containing 3:1 mix of 0.1 mm and 0.5 mm pre-sterilized glass beads (Sigma, St. Louis, MO, USA). Then 1 mL of a warm 60 °C lysis buffer (500 mM NaCl, 50 mM Tris-HCl, pH 8.0, 50 mM EDTA, 4% SDS) was added. The mixture was vortexed and homogenized with MiniLys (Bertin Technologies, France) for 3 min. The lysate was incubated at 70 °C for 15 min and centrifuged for 20 min at 14,000 rpm. The supernatant (1 mL) was transferred to the sterile tube and put on the ice. The pellet was added to a 1 mL of lysis buffer and the homogenization process was repeated. The supernatants were combined in the 15 mL tubes with the addition of 4 mL of 96% ethanol and 200 μL of 3 M sodium acetate. The mixture was incubated at −20 °C for not less than 1 h. The mixture was centrifuged for 15 min at 14,000 rpm at +4 °C, the supernatant was discarded, the DNA pellet was washed twice with 80% ethanol. The pellet was dried at 53 °C for 30–60 min and resuspended in 200 μL of sterilized milliQ water. The mixture was centrifuged and transferred into new tubes. Resulting DNA solution was treated with 10 μL of RNAse A (5 mg/mL) for 1 h at 37 °C, followed by additional round of chloroform purification. Chloroform was added to the solution in 1:1 ratio, tube was vortexed for 1 min and centrifuged at 5000× *g* for 5 min. Aqueous phase was transferred to new sterile tube and used for PCR dilutions. The obtained DNA solution was stored at −20 °C.

Amplicon sequencing of V4 variable region of microbial 16S rRNA gene was performed on a MiSeq sequencer (Illumina, San Diego, CA, USA) as described before [44].

### 4.6. Microbiome Data Processing

Raw microbiome data are available in the Sequence Read Archive (SRA) by the accession number PRJNA688839. The reads were processed using the Knomics-Biota system [45] (“16S dada2 Greengenes V4” pipeline based on the DADA2 algorithm and Greengenes database [46,47]) as previously described [48]. In the pipeline, the Greengenes database was preprocessed using TaxMan [49] based on the F515-R806 primers for V4 region of the 16S rRNA gene, and the sequences were clustered with 97% identity using cd-hit software version 4.8.1 [50]. For ambiguous sequences for which taxonomy could not be resolved based on the used primers, the slash (“/”) character was used (example: (Blautia/Dorea)). When the sequence could not be resolved at a particular taxonomic rank, the “_u” sign was used (referring to the term “unclassified”, example: “Lactobacillus_u”). There were minor changes to the original Knomics-Biota pipeline: the Chao1 index calculated on the level of ASVs (amplicon sequencing variants) after rarefaction to 3000 reads per sample was used to assess the alpha diversity; The beta diversity was estimated using Euclidean distance in Aitchison space [51].

### 4.7. Statistical Analysis

For all statistical procedures, the taxonomic data (unrarefied read counts per microbial taxon) were clr transformed; zero values were preliminarily imputed using Bayesian-multiplicative treatment implemented in the cmultRepl function in package zCompositions [52]. The metabolite concentrations data were log2 transformed after addition of 0.001 pseudocount.

Metabolic and gut microbial taxonomic profiles of samples from the CCI patients and healthy subjects were compared using the 1st time point per subject for the CCI patients with linear models. Taxonomic profiles were analyzed at different ranks—from order down to species. Adjustment for multiple comparison was applied separately for taxonomic and metabolic analysis results using Benjamini-Hochberg method. Patients with certain diagnosis were compared to all other patients during the analysis of diagnoses. The analysis was performed for all diagnoses except anoxic brain injury effort due to the small sample size in this group.

The associations of metabolic and taxonomic profiles with dynamics, neurological scales, diagnosis, infectious complication, therapy (antibiotics, type of nutrition) as well as between each other were assessed using mixed effect linear models. Measurements from all time points were used and patients ID was treated as a random effect in a model. Similar to the health status analysis, the taxonomic ranks from species to order were analyzed. Adjustments for multiple comparisons were applied separately within each metadata group (dynamics, neurological scales, therapy) and data type (taxonomic and metabolic) using Benjamini-Hochberg method. 

The taxonomic data associations with health status (CCI or healthy), dynamics and metabolic profiles were calculated with balances-based approach [23] as described previously [48]. The taxonomic rank for balances calculation was selected based on linear regression results as the rank at which the lowest adjusted *p*-value was observed. For health status analysis, the first time point measurements per patient were used and for the other analyses—the last point. We use threshold for the reproducibility of bacterial taxa > 50% and final model R^2^ > 0.2 to identify reliable associations using balances-based approach.

Differences in changes of microbial abundances and metabolite levels between the patients with different dynamics were assessed by ANCOVA approach, where dynamics were treated as an intercept. 

Alpha diversity associations with dynamics were assessed using mixed-effect linear models, where patient ID was treated as a random effect.

## 5. Conclusions

The CCI patients have a significant and patient-specific imbalance in the taxonomic composition of the gut microbiota. Pathophysiological role of an increased abundance of taxon associated with the biodegradation of tyrosine such as *Clostridiaceae*, *Coproccocus* and one of its metabolites (PhPA) found in patients with positive dynamics should be further confirmed in neurorehabilitation. It is possible that disrupted profile of the microbial metabolites of phenolic and other chemical classes plays an important role in neurological deficits. We are confident that CCI patients need microbiota-targeted therapies that can improve the efficacy of neurorehabilitation even in patients with severe brain damage. 

## Figures and Tables

**Table 1:** Concentrations of the microbial and mitochondrial metabolites (µM) and biomarkers in the serum samples of the CCI patients (*n* = 44) and healthy subjects (*n* = 20), Median (IQR 25–75%).

Metabolite	CCI Patients	Healthy Subjects *	FDR
Microbial Phenolic Metabolites µM			
PhLA	0.2 (0.1–0.4)	<LOD **	1.9 × 10^−7^
*p*-HPhAA	0.7 (0.3–1.8)	<LOD **	>0.05
*p*-HPhLA	0.8 (0.6–1.1)	0.7 (0.6–1.0)	>0.05
BA	0.5 (0.3–0.8)	0.4 (0.3–0.8)	>0.05
*p*-HBA	0.2 (0–0.9)	<LOD **	5.3 × 10^−10^
Mitochondrial Metabolites, µM			
Succinic Acid (SA)	6.3 (4.4–10.1)	22.0 (15.5–26.2)	6.3 × 10^−6^
Fumaric Acid (FA)	0.6 (0.3–1.2)	1.8 (1.3–2.4)	6.3 × 10^−6^
Biomarkers ***			
Procalcitonin	0.06 (0.03–0.09)	0.25 ng/mL	-
Interleukin-6	21 (12–33)	< 7 pg/mL	-
S100 protein	0.06 (0.04–0.1)	< 0.1 μg/L	-

* the results for the PhCA concentrations in healthy subjects were described previously [23]; ** the concentration is below the limit of detection (LOD); *** the reference values are provided as the levels of the biomarkers for the healthy subjects.

**Table 2:** Significant associations of the microbiome and metabolite profiles with neurological scales.

Taxon/Metabolite	Neurological Scales	Rank	*P* Value	FDR	Linear Model Coefficient
*(unclassified/Clostridiaceae/Lachnospiraceae)_u_u*	Rivermead Mobility Index	species	3.3 × 10^−6^	8.3 × 10^−4^	0.2962
*Ruminococcus (bromii/unclassified)*	Rivermead Mobility Index	species	4.0 × 10^−4^	3.6 × 10^−2^	0.2521
*(unclassified/Clostridiaceae/Lachnospiraceae)_u*	Rivermead Mobility Index	genus	4.4 × 10^−6^	8.8 × 10^−4^	0.2955
*(unclassified/Clostridiaceae/Lachnospiraceae)*	Rivermead Mobility Index	family	1.2 × 10^−5^	2.0 × 10^−3^	0.3200
*(unclassified/Clostridiaceae/Lachnospiraceae)_u_u*	Rankin	species	6.2 × 10^−7^	2.5 × 10^−4^	−1.0954
*(Coprococcus/unclassified)_u*	Rankin	species	1.0 × 10^−4^	1.4 × 10^−2^	−0.7768
*Ruminococcus (bromii/unclassified)*	Rankin	species	1.5 × 10^−4^	1.6 × 10^−2^	−0.9269
*(unclassified/Clostridiaceae/Lachnospiraceae)_u*	Rankin	genus	5.8 × 10^−7^	2.5 × 10^−4^	−1.1043
*(Coprococcus/unclassified)*	Rankin	genus	1.2 × 10^−4^	1.5 × 10^−2^	−0.7815
*Roseburia*	Rankin	genus	2.1 × 10^−4^	2.1 × 10^−2^	−0.7667
*(unclassified/Clostridiaceae/Lachnospiraceae)*	Rankin	family	7.6 × 10^−6^	2.5 × 10^−4^	−1.2307
PhPA	Rankin	-	1.0 × 10^−3^	3.4 × 10^−2^	−1.40
PhPA	GCS	-	7.6 × 10^−4^	3.4 × 10^−2^	0.65

**Table 3:** Reliable associations (the reproducibility of bacterial taxa > 50% and R^2^ > 0.2) between the metabolites levels and bacteria abundances identified using the balances-based approach (“+” direction denotes numerator and “-” direction-denominator).

Metabolite	Bacteria	Adjusted R^2^ of the Balance-Metabolite Model	Direction	Reproducibility of the Taxon
PhPA	*Coprococcus_u*	0.35593	+	78
S100	*Lactococcus_u*	0.20522	+	70
Succinic acid	*Bifidobacterium_u*	0.31986	+	60

**Table 4:** Significant associations between microbiome profiles and therapy in the CCI patients.

Taxon	Factor	Rank	*P* Value	FDR	Estimate
*(unclassified/Clostridium)_u*	antibiotics	species	1.2 × 10^−4^	0.0264	−1.77
*(unclassified/Clostridium)*	antibiotics	genus	1.0 × 10^−4^	0.0264	−1.79
*Ruminococcus (bromii/unclassified)*	nutrition type	species	3.0 × 10^−4^	0.0443	1.90
*[Eubacterium]_dolichum*	nutrition type	species	1.3 × 10^−4^	0.0264	−1.89

**Table 5:** Baseline clinical and laboratory characteristics of CCI patients upon admission to the ICU. Quantitative data are shown as median (1st and 3rd quartile); There is no significant differences between two groups of CCI patiets.

Parameters	Group with Positive Dynamics (*n* = 15)	Group with Negative Dynamics (*n* = 29)
Age	48 (29–59)	53 (39–64)
Number of patients taking antibiotics	6/15	15/29
Nutrition type	7/15	16/29
NIHSS	15 (11–19)	18 (13–24)
Rivermead Mobility Index	1 (0–8)	2 (0–3)
Rankin Scale	5 (4–5)	5 (4–5)
GCS	15 (14–15)	15 (13–15)
FOUR	14 (14–16)	14 (10–16)
PCT (0.25 ng/mL)	0.03 (0.02–0.06)	0.06 (0.03–0.1)
Sum of PhCA	3.2 (2.3–6.4)	4.1 (3.1–5.3)
S100 protein	0.05 (0.03–0.08)	0.06 (0.04–0.09)
IL-6	14.3 (12.8–27.6)	18.2 (12.9–31.9)

NIHSS—The National Institutes of Health Stroke Scale, or NIH Stroke Scale, Full Outline of UnResponsiveness (FOUR) scales.

## Data Availability

Raw microbiome data are available in the Sequence Read Archive (SRA) by the accession number PRJNA688839.

## Supplementary Materials

The following are available online at https://www.mdpi.com/2218-1989/11/2/122/s1, Table S1: Significant associations between microbiome profile and health status.

## References

[1] Nelson J.E., Cox C.E., Hope A.A., Carson S.S. (2010). Chronic critical illness. Am. J. Respir. Crit. Care Med..

[2] Cox C.E. (2012). Persistent systemic inflammation in chronic critical illness. Respir. Care.

[3] Parfenov A.L., Petrova M.V., Pichugina I.M., Luginina E.V. (2020). Comorbidity Development in Patients with Severe Brain Injury Resulting in Chronic Critical Condition. General Reanimatol..

[4] Giau V.V., Wu S.Y., Jamerlan A., Soo S.A., Kim A.N., Hulme J. (2018). Gut microbiota and their neuroinflammatory implications in Alzheimer’s disease. Nutrients.

[5] Kohler J., Borchers F., Endres M., Weiss B., Spies C., Emmrich J.V. (2019). Cognitive deficits following intensive care. Dtsch. Ärzteblatt Int..

[6] Ojima M., Motooka D., Shimizu K., Gotoh K., Shintani A., Yoshiya K., Nakamura S., Ogura H., Iida T., Shimazu T. (2016). Metagenomic analysis reveals dynamic changes of whole gut microbiota in the acute phase of intensive care unit patients. Dig. Dis. Sci..

[7] McDonald D., Ackermann G., Khailova L., Baird C., Heyland D., Kozar R., Lemieux M., Derenski K., King J., Vis-Kampen C. (2016). Extreme dysbiosis of the microbiome in critical illness. mSphere.

[8] Haak B.W., Levi M., Wiersinga W.J. (2017). Microbiota-targeted therapies on the intensive care unit. Curr. Opin. Crit. Care.

[9] Meng M., Klingensmith N.J., Coopersmith C.M. (2017). New insights into the gut as the driver of critical illness and organ failure. Curr. Opin. Crit. Care.

[10] Otani S., Coopersmith C.M. (2019). Gut integrity in critical illness. J. Intensive Care.

[11] Levy H., Hayes J., Boivin M., Tomba T. (2004). Transpyloric feeding tube placement in critically ill patients using electromyogram and erythromycin infusion. Chest.

[12] Luft V.M. (2010). Sovremennye vozmozhnosti nutricionnoj podderzhki bol’nyh v intensivnoj medicine. Vestn. Anesteziol. Reanimatol..

[13] Beloborodova N., Pautova A., Sergeev A., Fedotcheva N. (2019). Serum Levels of Mitochondrial and Microbial Metabolites Reflect Mitochondrial Dysfunction in Different Stages of Sepsis. Metabolites.

[14] Shenderov B.A., Sinitsa A.V., Zakharchenko M.M., Lang C. (2020). Metabolic Relationship Between the Host and Its Gut Microbiota. Metabiotics.

[15] Valerio F., Lavermicocca P., Pascale M., Visconti A. (2004). Production of phenyllactic acid by lactic acid bacteria: An approach to the selection of strains contributing to food quality and preservation. FEMS Microbiol. Lett..

[16] Beloborodova N.V., Khodakova A.S., Bairamov I.T., Olenin A.Y. (2009). Microbial origin of phenylcarboxylic acids in the human body. Biochemistry.

[17] Fedotcheva N.I., Kazakov R.E., Kondrashova M.N., Beloborodova N.V. (2008). Toxic effects of microbial phenolic acids on the functions of mitochondria. Toxicol. Lett..

[18] Beloborodova N.V., Moroz V.V., Bedova A.Y., Osipov A.A., Sarshor Y.N., Chernevskaya E.A. (2016). Participation of aromatic microbial metabolites in the development of severe infection and sepsis. Anesteziol. Reanimatol..

[19] Beloborodova N.V., Sarshor Y.N., Bedova A.Y., Chernevskaya E.A., Pautova A.K. (2018). Involvement of aromatic metabolites in the pathogenesis of septic shock. Shock.

[20] Moroz V.V., Beloborodova N.V., Osipov A.A., Vlasenko A.V., Bedova A.Y., Pautova A.K. (2016). Phenylcarboxylic acids in the assessment of the severity of patient condition and the efficiency of intensive treatment in critical care medicine. Gen. Reanimatol..

[21] Chernevskaya E., Beloborodova N., Klimenko N., Pautova A., Shilkin D., Gusarov V., Tyakht A. (2020). Serum and fecal profiles of aromatic microbial metabolites reflect gut microbiota disruption in critically ill patients: A prospective observational pilot study. Crit. Care.

[22] Cardoso S.M., Empadinhas N. (2018). The Microbiome-Mitochondria Dance in Prodromal Parkinson’s Disease. Front. Physiol..

[23] Pautova A.K., Bedova A.Y., Sarshor Y.N., Beloborodova N.V. (2018). Determination of aromatic microbial metabolites in blood serum by gas chromatography–mass spectrometry. J. Analyt. Chem..

[24] Rivera-Pinto J., Egozcue J.J., Pawlowsky-Glahn V., Paredes R., Noguera-Julian M., Calle M.L. (2018). Balances: A New Perspective for Microbiome Analysis. mSystems.

[25] Zaborin A., Smith D., Garfield K., Quensen J., Shakhsheer B., Kade M., Tirrell M., Tiedje J., Gilbert J.A., Zaborina O. (2014). Membership and behavior of ultra-low-diversity pathogen communities present in the gut of humans during prolonged critical illness. mBio.

[26] Ravi A., Halstead F.D., Bamford A., Casey A., Thomson N.M., Schaik W., Snelson C., Goulden R., Foster-Nyarko E., Savva G.M. (2019). Loss of microbial diversity and pathogen domination of the gut microbiota in critically ill patients. Microb. Genom..

[27] Li N., Wang X., Sun C., Wu X., Lu M., Si Y., Ye X., Wang T., Yu X., Zhao X. (2019). Change of intestinal microbiota in cerebral ischemic stroke patients. BMC Microbiol..

[28] Leslie D.L., Kozma L., Martin A., Landeros A., Katsovich L., King R.A., Leckman J.F. (2008). Neuropsychiatric disorders associated with streptococcal infection: A case-control study among privately insured children. J. Am. Acad. Child. Adolesc. Psychiatry.

[29] Lin P., Ding B., Feng C., Yin S., Zhang T., Qi X., Lv H., Guo X., Dong K., Zhu Y. (2017). Prevotella and Klebsiella proportions in fecal microbial communities are potential characteristic parameters for patients with major depressive disorder. J. Affect. Disord..

[30] Bo J., Wang S., Bi Y., Ma S., Wang M., Du Z. (2020). Eggerthella lenta bloodstream infections: Two cases and review of the literature. Future Microbiol..

[31] Rekdal V.M., Bess E.N., Bisanz J.E., Turnbaugh P.J., Balskus E.P. (2019). Discovery and inhibition of an interspecies gut bacterial pathway for Levodopa metabolism. Science.

[32] Ticinesi A., Mancabelli L., Tagliaferri S., Nouvenne A., Milani C., Del Rio D., Lauretani F., Maggio M.G., Ventura M., Meschi T. (2020). The Gut-Muscle Axis in Older Subjects with Low Muscle Mass and Performance: A Proof of Concept Study Exploring Fecal Microbiota Composition and Function with Shotgun Metagenomics Sequencing. Int. J. Mol. Sci..

[33] Muñiz Pedrogo D.A., Chen J., Hillmann B., Jeraldo P., Al-Ghalith G., Taneja V., Davis J.M., Knights D., Nelson H., Faubion W.A. (2019). An Increased Abundance of Clostridiaceae Characterizes Arthritis in Inflammatory Bowel Disease and Rheumatoid Arthritis: A Cross-sectional Study. Inflamm. Bowel Dis..

[34] Dai Z.L., Wu G., Zhu W.Y. (2011). Amino acid metabolism in intestinal bacteria: Links between gut ecology and host health. Front. Biosci..

[35] Geerlings S.Y., Kostopoulos I., de Vos W.M., Belzer C. (2018). Akkermansia muciniphila in the human gastrointestinal tract: When, where, and how?. Microorganisms.

[36] Jangi S., Gandhi R., Cox L.M., Li N., von Glehn F., Yan R., Patel B., Mazzola M.A., Liu S., Glanz B.L. (2016). Alterations of the human gut microbiome in multiple sclerosis. Nat. Commun..

[37] Toma I., Kang J.J., Sipos A., Vargas S., Bansal E., Hanner F., Meer E., Peti-Peterdi J. (2008). Succinate receptor GPR91 provides a direct link between high glucose levels and renin release in murine and rabbit kidney. J. Clin. Investig..

[38] Nguyen T.D., Prykhodko O., Fåk Hållenius F., Nyman M. (2019). Monovalerin and trivalerin increase brain acetic acid, decrease liver succinic acid, and alter gut microbiota in rats fed high-fat diets. Eur. J. Nutr..

[39] Valles-Colomer M., Falony G., Darzi Y., Tigchelaar E.F., Wang J., Tito R.Y., Schiweck C., Kurilshikov A., Joossens M., Wijmenga C. (2019). The neuroactive potential of the human gut microbiota in quality of life and depression. Nat. Microbiol..

[40] Chernevskaya E.A., Meglei A.Y., Buyakova I.V., Kovaleva N.Y., Gorshkov K.M., Zakharchenko V.E., Beloborodova N.V. (2020). Taxonomic dysbiosis of gut microbiota and serum biomarkers reflect severity of central nervous system injury. Bull. RSMU.

[41] Liu B., Zhu X., Cui Y., Wang W., Liu H., Li Z., Guo Z., Ma S., Li D., Wang C. (2021). Consumption of Dietary Fiber from Different Sources during Pregnancy Alters Sow Gut Microbiota and Improves Performance and Reduces Inflammation in Sows and Piglets. mSystems.

[42] Iablokov S.N., Klimenko N.S., Efimova D.A., Shashkova T., Novichkov P.S., Rodionov D.A., Tyakht A.V. (2020). Metabolic phenotypes as potential biomarkers for linking gut microbiome with inflammatory bowel diseases. Front. Mol. Biosci..

[43] Saver J.L., Filip B., Hamilton S., Yanes A., Craig S., Cho M., Conwit R., Starkman S. (2010). Improving the reliability of stroke disability grading in clinical trials and clinical practice: The Rankin Focused Assessment (RFA). Stroke.

[44] Klimenko N.S., Tyakht A.V., Popenko A.S., Vasiliev A.S., Altukhov I.A., Ischenko D.S., Shashkova T.I., Efimova D.A., Nikogosov D.A., Osipenko D.A. (2018). Microbiome Responses to an Uncontrolled Short-Term Diet Intervention in the Frame of the Citizen Science Project. Nutrients.

[45] Efimova D., Tyakht A., Popenko A., Vasilyev A., Altukhov I., Dovidchenko N., Odintsova V., Klimenko N., Loshkarev R., Pashkova M. (2018). Knomics-Biota—A system for exploratory analysis of human gut microbiota data. BioData Min..

[46] Callahan B.J., McMurdie P.J., Rosen M.J., Han A.W., Johnson A.J.A., Holmes S.P. (2016). DADA2: High-Resolution Sample Inference from Illumina Amplicon Data. Nat. Methods.

[47] DeSantis T.Z., Hugenholtz H., Larsen N., Rojas M., Brodie E.L., Keller K., Huber T., Dalevi D., Hu P., Andersen G.L. (2006). Greengenes, a Chimera-Checked 16S rRNA Gene Database and Workbench Compatible with ARB. Appl. Environ. Microbiol..

[48] Andrianova N.V., Popkov V.A., Klimenko N.S., Tyakht A.V., Baydakova G.V., Frolova O.Y., Zorova L.D., Pevzner I.B., Zorov D.B., Plotnikov E.Y. (2020). Microbiome-Metabolome Signature of Acute Kidney Injury. Metabolites.

[49] Brandt B.W., Bonder M.J., Huse S.M., Zaura E. (2012). TaxMan: A Server to Trim rRNA Reference Databases and Inspect Taxonomic Coverage. Nucleic Acids Res..

[50] Fu L., Niu B., Zhu Z., Wu S., Li W. (2012). CD-HIT: Accelerated for Clustering the next-Generation Sequencing Data. Bioinformatics.

[51] Aitchison J. (1982). The Statistical Analysis of Compositional Data. J. R. Stat. Soc..

[52] Martín-Fernández J.-A., Hron K., Templ M., Filzmoser P., Palarea-Albaladejo J. (2015). Bayesian-Multiplicative Treatment of Count Zeros in Compositional Data Sets. Stat. Model..

