# Supplementary material for: Host-Microbiome Interactions Mediated by Phenolic Metabolites in Chronically Critically Ill Patients
Source: Metabolites. 2021 Feb 20;11(2):122. doi: 10.3390/metabo11020122 (PMC7924600; doi:10.3390/metabo11020122)
Supplement: Supplementary file 1 [file metabolites-11-00122-s001.pdf]

metabolites

MDPI

Table S1. Significant associations between microbiome profile and health status.

Taxon	*p* Value	Linear Model Coefficient	Taxonomic Rank	FDR
k__Bacteria;p__Actinobacteria;c__Coriobacteriia;o__Coriobacteriales;f__Coriobacteriaceae;g__;s__	0	−0.0042	sp	0.0001
k__Bacteria;p__Bacteroidetes;c__Bacteroidia;o__Bacteroidales;f__Prevotellaceae;g__Prevotella;s__	0.0002	−0.0141	sp	0.0009
k__Bacteria;p__Bacteroidetes;c__Bacteroidia;o__Bacteroidales;f__S24-7;g__;s__	0.0006	−0.0112	sp	0.0028
k__Bacteria;p__Bacteroidetes;c__Bacteroidia;o__Bacteroidales;f__[Barnesiellaceae];g__;s__	0.0094	−0.0009	sp	0.0319
k__Bacteria;p__Bacteroidetes;c__Bacteroidia;o__Bacteroidales;f__[Paraprevotellaceae];g__Paraprevotella;s__	0	−0.0016	sp	0
k__Bacteria;p__Cyanobacteria;c__4C0d-2;o__YS2;f__;g__;s__	0.0029	−0.0009	sp	0.0112
k__Bacteria;p__Firmicutes;c__Clostridia;o__Clostridiales;f__;g__;s__	0	−0.097	sp	0.0002
k__Bacteria;p__Firmicutes;c__Clostridia;o__Clostridiales;f__(Christensenellaceae/Ruminococcaceae/Clostridiaceae);g__;s__	0	−0.0035	sp	0.0002
k__Bacteria;p__Firmicutes;c__Clostridia;o__Clostridiales;f__(Clostridiaceae/Ruminococcaceae);g__;s__	0	−0.0015	sp	0
k__Bacteria;p__Firmicutes;c__Clostridia;o__Clostridiales;f__(Clostridiaceae/[Mogibacteriaceae]/Peptostreptococcaceae);g__;s__	0.0075	−0.0005	sp	0.0258
k__Bacteria;p__Firmicutes;c__Clostridia;o__Clostridiales;f__(Lachnospiraceae/Ruminococcaceae/unclassified);g__;s__	0	−0.0032	sp	0.0001
k__Bacteria;p__Firmicutes;c__Clostridia;o__Clostridiales;f__(Ruminococcaceae/Clostridiaceae/unclassified);g__;s__	0	−0.0068	sp	0
k__Bacteria;p__Firmicutes;c__Clostridia;o__Clostridiales;f__(unclassified/Clostridiaceae/Lachnospiraceae);g__;s__	0	−0.0103	sp	0.0001
k__Bacteria;p__Firmicutes;c__Clostridia;o__Clostridiales;f__Christensenellaceae;g__;s__	0	−0.0066	sp	0.0001
k__Bacteria;p__Firmicutes;c__Clostridia;o__Clostridiales;f__Lachnospiraceae;g__(Coprococcus/unclassified);s__	0	−0.0108	sp	0
k__Bacteria;p__Firmicutes;c__Clostridia;o__Clostridiales;f__Lachnospiraceae;g__(unclassified/Blautia);s__	0.0001	−0.0014	sp	0.0006
k__Bacteria;p__Firmicutes;c__Clostridia;o__Clostridiales;f__Lachnospiraceae;g__(unclassified/Roseburia/Blautia);s__	0	−0.0297	sp	0
k__Bacteria;p__Firmicutes;c__Clostridia;o__Clostridiales;f__Lachnospiraceae;g__Coprococcus;s__	0.0011	−0.0109	sp	0.0045
k__Bacteria;p__Firmicutes;c__Clostridia;o__Clostridiales;f__Lachnospiraceae;g__Lachnospira;s__	0	−0.0138	sp	0
k__Bacteria;p__Firmicutes;c__Clostridia;o__Clostridiales;f__Lachnospiraceae;g__Roseburia;s__faecis	0	−0.0129	sp	0.0001
k__Bacteria;p__Firmicutes;c__Clostridia;o__Clostridiales;f__Ruminococcaceae;g__(unclassified/Oscillospira);s__	0.0001	−0.032	sp	0.0003
k__Bacteria;p__Firmicutes;c__Clostridia;o__Clostridiales;f__Ruminococcaceae;g__Faecalibacterium;s__prausnitzii	0	−0.147	sp	0
k__Bacteria;p__Firmicutes;c__Clostridia;o__Clostridiales;f__Ruminococcaceae;g__Ruminococcus;s__(bromii/unclassified)	0.0011	−0.0139	sp	0.0045
k__Bacteria;p__Firmicutes;c__Clostridia;o__Clostridiales;f__Veillonellaceae;g__Dialister;s__	0.0005	−0.0223	sp	0.0021
k__Bacteria;p__Tenericutes;c__Mollicutes;o__RF39;f__;g__;s__	0	−0.0053	sp	0
k__Bacteria;p__Actinobacteria;c__Coriobacteriia;o__Coriobacteriales;f__Coriobacteriaceae;g__	0	−0.004	g	0.0001

k__Bacteria;p__Bacteroidetes;c__Bacteroidia;o__Bacteroidales;f__Prevotellaceae;g__Prevotella	0.0014	−0.031	g	0.0056
k__Bacteria;p__Bacteroidetes;c__Bacteroidia;o__Bacteroidales;f__S24-7;g__	0.0007	−0.0111	g	0.003
k__Bacteria;p__Bacteroidetes;c__Bacteroidia;o__Bacteroidales;f__[Barnesiellaceae];g__	0.0099	−0.0009	g	0.0322
k__Bacteria;p__Bacteroidetes;c__Bacteroidia;o__Bacteroidales;f__[Paraprevotellaceae];g__Paraprevotella	0	−0.0015	g	0
k__Bacteria;p__Cyanobacteria;c__4C0d-2;o__YS2;f__;g__	0.003	−0.0009	g	0.0114
k__Bacteria;p__Firmicutes;c__Clostridia;o__Clostridiales;f__;g__	0	−0.0947	g	0.0001
k__Bacteria;p__Firmicutes;c__Clostridia;o__Clostridiales;f__(Christensenellaceae/Ruminococcaceae/Clostridiaceae);g__	0	−0.0034	g	0.0002
k__Bacteria;p__Firmicutes;c__Clostridia;o__Clostridiales;f__(Clostridiaceae/Ruminococcaceae);g__	0	−0.0015	g	0
k__Bacteria;p__Firmicutes;c__Clostridia;o__Clostridiales;f__(Clostridiaceae/[Mogibacteriaceae]/Peptostreptococcaceae);g__	0.0031	−0.0005	g	0.0115
k__Bacteria;p__Firmicutes;c__Clostridia;o__Clostridiales;f__(Lachnospiraceae/Ruminococcaceae/unclassified);g__	0	−0.0032	g	0.0001
k__Bacteria;p__Firmicutes;c__Clostridia;o__Clostridiales;f__(Ruminococcaceae/Clostridiaceae/unclassified);g__	0	−0.0067	g	0
k__Bacteria;p__Firmicutes;c__Clostridia;o__Clostridiales;f__(unclassified/Clostridiaceae/Lachnospiraceae);g__	0	−0.01	g	0.0001
k__Bacteria;p__Firmicutes;c__Clostridia;o__Clostridiales;f__Christensenellaceae;g__	0	−0.0064	g	0.0001
k__Bacteria;p__Firmicutes;c__Clostridia;o__Clostridiales;f__Lachnospiraceae;g__(Coprococcus/unclassified)	0	−0.0105	g	0
k__Bacteria;p__Firmicutes;c__Clostridia;o__Clostridiales;f__Lachnospiraceae;g__(unclassified/Blautia)	0.0001	−0.0014	g	0.0006
k__Bacteria;p__Firmicutes;c__Clostridia;o__Clostridiales;f__Lachnospiraceae;g__(unclassified/Roseburia/Blautia)	0	−0.0289	g	0
k__Bacteria;p__Firmicutes;c__Clostridia;o__Clostridiales;f__Lachnospiraceae;g__Coprococcus	0	−0.0151	g	0.0002
k__Bacteria;p__Firmicutes;c__Clostridia;o__Clostridiales;f__Lachnospiraceae;g__Lachnospira	0	−0.0137	g	0
k__Bacteria;p__Firmicutes;c__Clostridia;o__Clostridiales;f__Lachnospiraceae;g__Roseburia	0.0007	−0.0165	g	0.0031
k__Bacteria;p__Firmicutes;c__Clostridia;o__Clostridiales;f__Ruminococcaceae;g__(unclassified/Oscillospira)	0	−0.0314	g	0.0002
k__Bacteria;p__Firmicutes;c__Clostridia;o__Clostridiales;f__Ruminococcaceae;g__Faecalibacterium	0	−0.1437	g	0
k__Bacteria;p__Firmicutes;c__Clostridia;o__Clostridiales;f__Ruminococcaceae;g__Ruminococcus	0	−0.0271	g	0.0002
k__Bacteria;p__Firmicutes;c__Clostridia;o__Clostridiales;f__Veillonellaceae;g__Dialister	0.0004	−0.0218	g	0.002
k__Bacteria;p__Tenericutes;c__Mollicutes;o__RF39;f__;g__	0	−0.0052	g	0
k__Bacteria;p__Bacteroidetes;c__Bacteroidia;o__Bacteroidales;f__Prevotellaceae	0.0013	−0.0298	f	0.0052
k__Bacteria;p__Bacteroidetes;c__Bacteroidia;o__Bacteroidales;f__S24-7	0.0007	−0.0107	f	0.003
k__Bacteria;p__Bacteroidetes;c__Bacteroidia;o__Bacteroidales;f__[Barnesiellaceae]	0.0103	−0.0009	f	0.0329
k__Bacteria;p__Bacteroidetes;c__Bacteroidia;o__Bacteroidales;f__[Paraprevotellaceae]	0.0001	−0.0057	f	0.0004
k__Bacteria;p__Cyanobacteria;c__4C0d-2;o__YS2;f__	0.0033	−0.0009	f	0.0119
k__Bacteria;p__Firmicutes;c__Clostridia;o__Clostridiales;f__	0	−0.0928	f	0.0001
k__Bacteria;p__Firmicutes;c__Clostridia;o__Clostridiales;f__(Christensenellaceae/Ruminococcaceae/Clostridiaceae)	0	−0.0033	f	0.0002
k__Bacteria;p__Firmicutes;c__Clostridia;o__Clostridiales;f__(Clostridiaceae/Ruminococcaceae)	0	−0.0014	f	0

k__Bacteria;p__Firmicutes;c__Clostridia;o__Clostridiales;f__(Clostridiaceae/[Mogibacteriaceae]/Peptostreptococcaceae)	0.0035	−0.0005	f	0.0124
k__Bacteria;p__Firmicutes;c__Clostridia;o__Clostridiales;f__(Lachnospiraceae/Ruminococcaceae/unclassified)	0	−0.003	f	0.0001
k__Bacteria;p__Firmicutes;c__Clostridia;o__Clostridiales;f__(Ruminococcaceae/Clostridiaceae/unclassified)	0	−0.0066	f	0
k__Bacteria;p__Firmicutes;c__Clostridia;o__Clostridiales;f__(unclassified/Clostridiaceae/Lachnospiraceae)	0	−0.0097	f	0.0001
k__Bacteria;p__Firmicutes;c__Clostridia;o__Clostridiales;f__Ruminococcaceae	0	−0.2324	f	0
k__Bacteria;p__Tenericutes;c__Mollicutes;o__RF39;f__	0	−0.005	f	0
k__Bacteria;p__Cyanobacteria	0.004	−0.0009	p	0.014
k__Bacteria;p__Firmicutes	0.0001	−0.3012	p	0.0005
k__Bacteria;p__Tenericutes	0	−0.005	p	0
k__Bacteria;p__Cyanobacteria;c__4C0d-2	0.0031	−0.0009	c	0.0114
k__Bacteria;p__Firmicutes;c__Bacilli	0.0096	0.1492	c	0.0319
k__Bacteria;p__Firmicutes;c__Clostridia	0	−0.4475	c	0
k__Bacteria;p__Tenericutes;c__Mollicutes	0	−0.005	c	0
k__Bacteria;p__Cyanobacteria;c__4C0d-2;o__YS2	0.0032	−0.0009	o	0.0115
k__Bacteria;p__Firmicutes;c__Bacilli;o__Lactobacillales	0.0098	0.149	o	0.0322
k__Bacteria;p__Firmicutes;c__Clostridia;o__Clostridiales	0	−0.4477	o	0
k__Bacteria;p__Tenericutes;c__Mollicutes;o__RF39	0	−0.005	o	0
